# Conformational fingerprint of blood and tissue ACEs: Personalized approach

**DOI:** 10.1371/journal.pone.0209861

**Published:** 2018-12-27

**Authors:** Sergei M. Danilov, Victoria E. Tikhomirova, Olga V. Kryukova, Alexander V. Balatsky, Naida I. Bulaeva, Elena Z. Golukhova, Leo A. Bokeria, Larisa M. Samokhodskaya, Olga A. Kost

**Affiliations:** 1 Department of Anesthesiology, University of Illinois at Chicago, Illinois, United States of America; 2 University of Arizona Health Sciences, Tucson, Arizona, United States of America; 3 Medical Center, Lomonosov Moscow State University, Russia; 4 Chemistry Faculty, Lomonosov Moscow State University, Russia; 5 Bakulev Center for Cardiovascular Surgery, Moscow, Russia; Duke University School of Medicine, UNITED STATES

## Abstract

**Background:**

The pattern of binding of monoclonal antibodies (mAbs) to 18 epitopes on human angiotensin I-converting enzyme (ACE)–“conformational fingerprint of ACE”–is a sensitive marker of subtle conformational changes of ACE due to mutations, different glycosylation in various cells, the presence of ACE inhibitors and specific effectors, etc.

**Methodology/Principal findings:**

We described in detail the methodology of the conformational fingerprinting of human blood and tissue ACEs that allows detecting differences in surface topography of ACE from different tissues, as well detecting inter-individual differences. Besides, we compared the sensitivity of the detection of ACE inhibitors in the patient’s plasma using conformational fingerprinting of ACE (with only 2 mAbs to ACE, 1G12 and 9B9) and already accepted kinetic assay and demonstrated that the mAbs-based assay is an order of magnitude more sensitive. This approach is also very effective in detection of known (like bilirubin and lysozyme) and still unknown ACE effectors/inhibitors which nature and set could vary in different tissues or different patients.

**Conclusions/Significance:**

Phenotyping of ACE (and conformational fingerprinting of ACE as a part of this novel approach for characterization of ACE) in individuals really became informative and clinically relevant. Appreciation (and counting on) of inter-individual differences in ACE conformation and accompanying effectors make the application of this approach for future personalized medicine with ACE inhibitors more accurate. This (or similar) methodology can be applied to any enzyme/protein for which there is a number of mAbs to its different epitopes.

## Introduction

Angiotensin I-converting enzyme (ACE, CD143, EC 3.4.15.1), a zinc-metallopeptidase, is a key regulator of blood pressure participating in the development of vascular pathology and remodeling. The somatic isoform of ACE is highly expressed as a type-I transmembrane glycoprotein in endothelial, some epithelial, as well as macrophage and dendritic cells. Somatic ACE also presents as a soluble form, e.g., in plasma, cerebrospinal and seminal fluids, which lacks the transmembrane domain responsible for membrane attachment (for review see [1–2]).

Two homologous domains (N and C domains) within a single polypeptide chain comprise the majority of the structure of ACE, each containing a functional active center [3]. The three-dimensional crystal structure of ACE is still unknown. However, the model of the two-domain ACE has been recently suggested, based on the solved crystal structures of the individual C and N domains, epitope mapping of monoclonal antibodies (mAbs) to ACE, and on the electron microscopy picture of pig somatic ACE [4].

Recent ACE studies with mAbs recognizing different conformational epitopes on the surface of the catalytically active N domain (eight mAbs) and the C domain (eight mAbs) of human ACE molecule revealed that the pattern of mAb binding to ACE is potentially a very sensitive marker of the local conformation of ACE globule [5]. The changes of this pattern could be definitely attributed to the changes of the topography of the epitopes for the distinct mAbs due to denaturation of ACE globule, chemical modification, mutations, or the binding of inhibitors, as well as protein and low molecular weight (LMW) effectors [5–9]. It is noteworthy that ACE contains 17 potential N-glycosylation sites and the epitopes of all mAbs contain n at least one site. As ACE glycosylation could be both cell- and tissue-specific due to different post-translational modification of ACE globule in different cells, the local topography of ACE surface produced by different cells could be also unique. We demonstrated previously that the pattern of ACE binding by a set of mAbs to 16 epitopes of human ACE–"conformational fingerprint of ACE"–is indeed cell- and tissue-specific [5, 9–10] and, moreover, confirmed that tissue-specific glycosylation of ACE is an important structural requirement for this specific “conformational fingerprint” [10–12].

Here, we described in detail a methodology of “conformational fingerprinting” of ACE using a panel of monoclonal antibodies to this enzyme. This approach allows one to detect the presence of ACE inhibitors in the patient’s blood and provides valuable information on the presence of low-molecular weight (LMW) effectors and ACE-binding proteins in the plasma [8] and this study and tissues (this study). Moreover, we demonstrated that conformational fingerprinting of ACE is a sensitive potential tool for detection of even inter-individual differences in ACE conformation.

We believe that similar approach could be applied to any enzyme/protein for which there is a number of mAbs to its different epitopes. This approach could provide unique information about an enzyme in question and its effectors, not available by any other method.

## Materials and methods

### ACEs from different sources

The work was carried out in accordance with The Code of Ethics of World Medical Association (Declaration of Helsinki) and was approved by the Institutional Review Boards of the Bakulev Center of Cardiovascular Surgery, Moscow University and the University of Illinois at Chicago. None of the donors were from the vulnerable population and all donors or next of kin provided written informed consent that was freely given. Human citrated plasma and serum, seminal fluid, lung and heart tissue homogenates were used as sources of somatic, two-domain ACE. Human plasma was used as individual plasma samples from different donors as well as a pool from 80 donors. Seminal fluid was pooled from ejaculates of more than 30 individuals. Heart and lung homogenates were prepared from tissues of individual donors as described [9] using 1:9 (weight: volume of PBS) ratio. Previously we found that ACE activity in tissue homogenates (or cell lysates) prepared with 0.25% Triton X-100 do not tolerate long storage as frozen samples (unpublished). In this study, we used freshly prepared homogenate, no proteinase or sialidase inhibitors were added.

ACEs were purified from corresponding sources using anion-exchange chromatography on DEAE-Toyopearl 650M and lisinopril affinity chromatography as in [13], then further washed and concentrated on Vivaspin filtration membranes (GE Healthcare, Sartorius Corp., Bohemia, NY) with 100 kDa limit as in [9]. Recombinant soluble ACE was obtained from culture fluid of CHO cells transfected with ACE lacking transmembrane anchor [14], kindly provided by F. Alhenc-Gelas (then INSERM Unit 352, Paris, France).

### ACE activity assay

ACE activity in blood serum/plasma, seminal fluid or homogenates of human organs was measured using a fluorimetric assay with two ACE substrates, 2 mM Z-Phe-His-Leu (ZPHL) and 5 mM Hip-His-Leu (HHL), at pH 8.3 [15]. Briefly, 20–40 μl aliquots of samples diluted in PBS-BSA (0.1 mg/ml) were added to 200 μl of ACE substrate and incubated for an appropriate time at 37° C. The His-Leu product was quantified fluorimetrically via complexing with *o*-phtaldialdehyde.

### Immunological characterization of ACE (Plate immunoprecipitation assay)

Ninety six-well plates (high binding, Corning Inc., Corning, NY, USA) were coated with anti-ACE mAbs via goat anti-mouse IgG (Pierce, Rockford, IL, USA or IMTEK, Moscow, Russia) bridge [16] and incubated with different sources of ACE, which were equilibrated for ACE activity with ZPHL as a substrate. After washing off unbound ACE, plate-bound ACE activity was measured by adding a substrate for ACE (ZPHL) directly into the wells [16]. Sixteen mAbs to human ACE were generated in our lab [5], while mAb BB9 [17] to the N domain of ACE was kindly provided by Paul J Simmons (then Brown Foundation of Molecular Medicine, University of Texas Health Science Center, Houston, TX, USA). Additional mAb to ACE (clone 2H4 to the N domain of ACE) was generated in collaboration with Ilya N. Trakht and Gavreel F. Kalantarov (Columbia University, New York, NY, USA) (unpublished). For most experiments, we used a panel of 16 mAbs that were generated in our lab, while in some we tested also two new mAbs.

### Sequencing and genotyping

Genomic DNA was obtained from heart tissue of patients #11 and # 27 by QIAamp DNA Mini Kit (Qiagen, Hilden, Germany), and 9 exons of ACE gene (14th–20^th^, 23^th^ and 25^th^) were sequenced, using primers designed by Dufour et al. [18] as well from other sources (listed in Table 1). Briefly, PCR was made under the following conditions: 95°C for 5 min and then 40 cycles including melting at 95°C for 10 s, annealing at T_annealing_ (listed in Table 1) for 10 s and elongation at 72°C for 20 s. PCR products were separated in 1% agarose gel), isolated by QIAquick Gel Extraction Kit (Qiagen) and sequenced using the same primers, BigDye Terminator v3.1 Cycle Sequencing Kit (Thermo Fischer Scientific, Waltham, MA, USA) and 3730xl DNA Analyzer (Applied Biosystems, Waltham, MA, USA).

**Table 1:** Primers used for sequencing.

Exon	Sequence	Source	T_annealing_
14	GCAGAGGTTTGTCTGTTTCC TGTGTATGCACATGCTCAGG	[18]	56°C
15&16	AGCCCTCAGCTCCCACTTG CTCTGTGCTCTCACCCAGC	[18]	56°C
17	TCCCATCCTTTCTCCCATTTC ACGTAGGCATGCAGGTTGAGG	SeqWright, Inc. (Houston, TX)	56°C
18	AACATCACTGGCACTTGGGT AGATCTGCAGTGAATGTGGCA	In-house designed	56°C
19	GGACTGGGATCTGGAGCGC TCCTGAATCAGAGGGTCCCTCC	In-house designed	64°C
20	TGTCTTTCCTCTCTCTGCCGTC CACTCAGTCCCAAACTCTGGC	SeqWright, Inc. (Houston, TX)	56°C
23	CAGCCTGGTTCTCCCCAAACTC AAACACAAAGCTGTGGGTACTGCC	In-house designed	65°C
25	CCATGTCCTTCTGACTCTG TCCGAGGTCAGCCGCATGG	[18]	56°C

### Statistical analysis

All data are means ± SEM. Significance was analyzed using the Mann-Whitney test with STATISTICA 6 (StatSoft, Inc., OK).

## Results and discussion

### Enzyme (ACE) immune-capture assay

**Fig 1:**
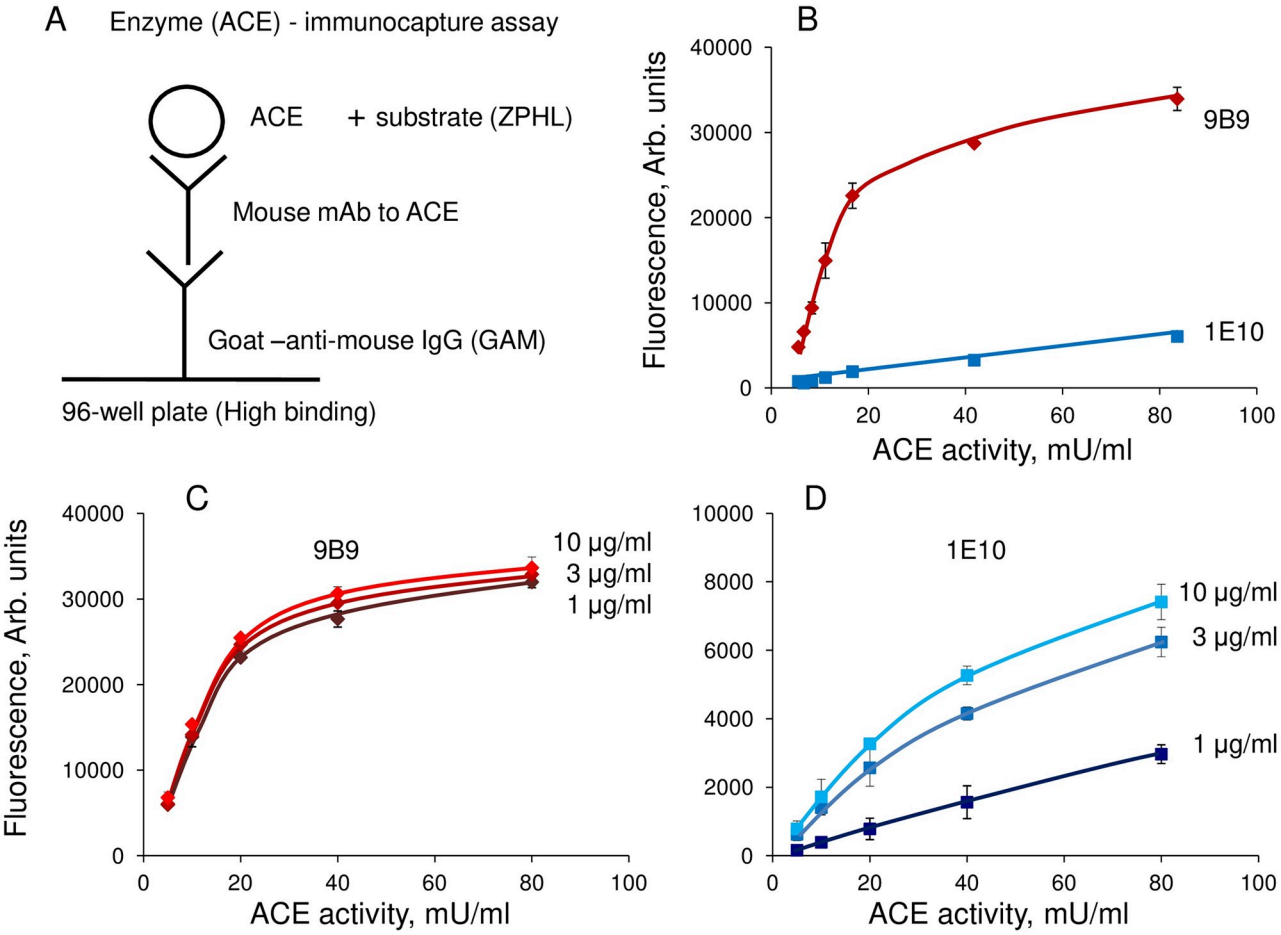
Enzyme (ACE) immune-capture assay. **A**. Scheme of the method. Goat anti-mouse IgG are loaded on the 96-wells plate to minimize non-specific adsorption of both mAbs and ACE, as well as prevent putative denaturation of some mAbs as a result of a contact with plastic. After washing of unbound anti-mouse IgG, mAbs to ACE are applied to the plate and, after another washing, analyzed ACE source is added. The amount of ACE in complex with any mAb is estimated by measuring precipitated ACE activity towards specific substrate, usually ZPHL, added directly into the wells. **B**. The dependence of the fluorescence (reflecting relative ACE activity in wells) on the loaded ACE activity on wells covered by strong mAb 9B9 (10 μg/ml) and weak mAb 1E10 (10 μg/ml). **C**. The dependence of the fluorescence signal on the loaded ACE activity at different concentrations of the loaded strong mAb 9B9. **D**. The dependence of the fluorescence signal on the loaded ACE activity at different concentrations of the loaded weak mAb 1E10.

In order to quantify the amount of immunoreactive ACE protein and to analyze the changes in the local ACE surface conformation we used enzyme (ACE) immune-capture assay with panel of mAbs to different epitopes on the surface of N- and C domains of ACE [16, 19]. The scheme illustrating the method is presented on the Fig 1A. High-binding 96-well plate was coated with different mAbs to ACE via goat-anti-mouse IgG bridge (10 μg/ml, 50 μl in each well) in PBS. After washing of unbound mAbs the source of ACE was added (usually 2–20 mU/ml) and then, after overnight incubation and washing of unbound ACE, substrate for ACE (usually 1 mM ZPHL in 100 mM phosphate buffer, pH 8.3, containing 300 mM NaCl,) was added and precipitated ACE activity was estimated directly in the wells [16]. Generally, the linear relation of added ACE and precipitated ACE activity in wells (the more loaded ACE activity—the more precipitated ACE activity) was observed with not more than 20 mU /ml of loaded ACE activity especially when high-binding mAb, e.g., mAb 9B9, was used (Fig 1B–1D). In order to save valuable mAbs, we used not more than 3 μg/ml of pure mAbs (or 1/20 dilution of hybridoma cell culture medium) which was found to be sufficient for coating (Fig 1C and 1D). Usually the amount of ACE immunoreactive protein was estimated using the strongest mAb to ACE, clone 9B9 [16, 20], while a pattern of ACE activity precipitation by a panel of mAbs to different epitopes on ACE—conformational fingerprint of ACE—was used for the estimation of local conformational differences in ACE surface topography due to disease [5, 7, 21] or due to tissue origin of ACE [5, 9, 10].

**Fig 2:**
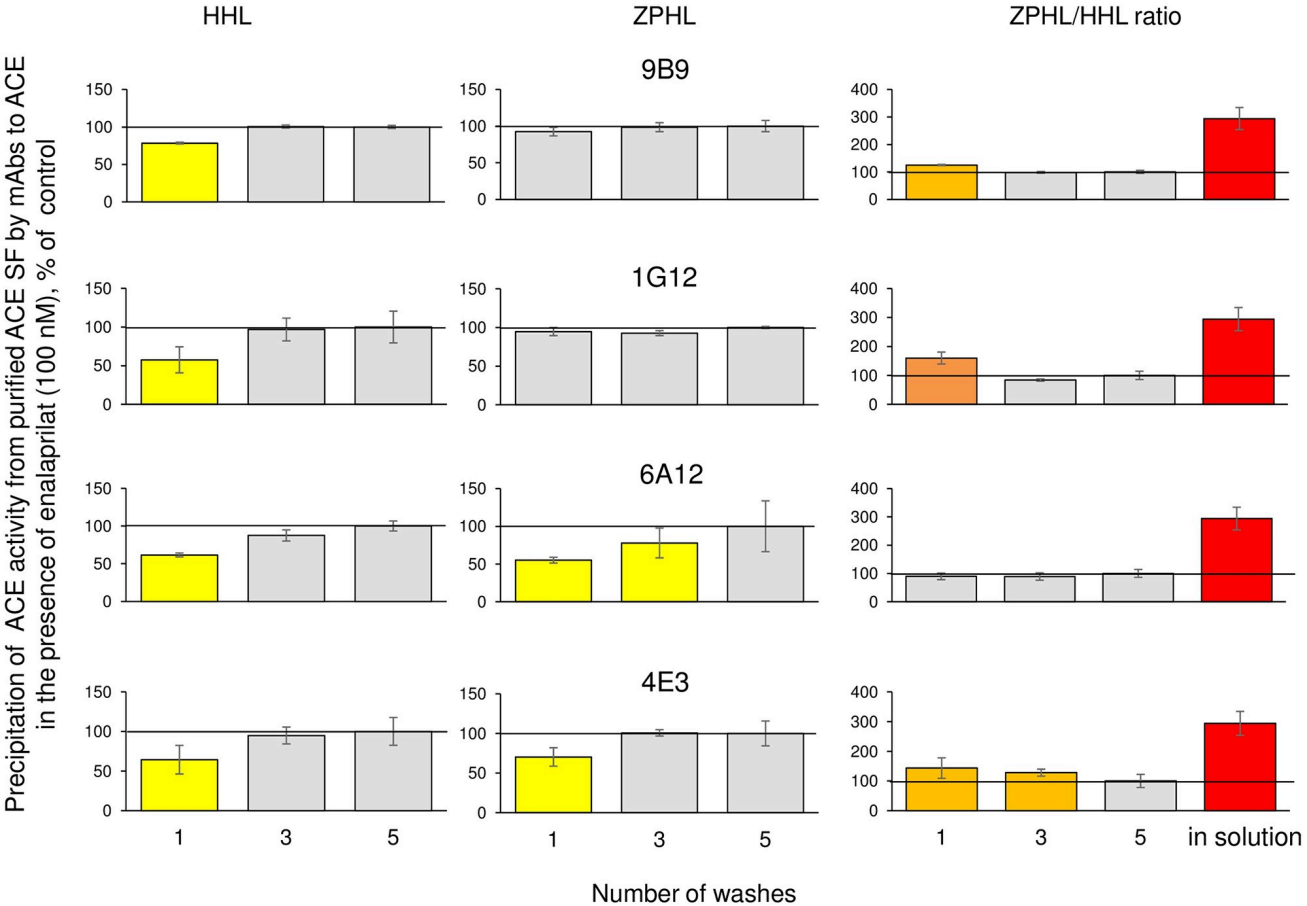
Effect of plate washing on the dissociation of ACE inhibitor from ACE. The residual ACE activity towards substrates, HHL and ZPHL, as well the ratio of the rates of the hydrolysis of these substrates, ZPHL/HHL ratio, were determined in the wells coated by goat anti-mouse IgG, four mAbs to ACE (strong and weak) and, then, the mixture of purified seminal fluid ACE with inhibitor enalaprilat (100 nM) after different number of washings with water with 0.05% (v/v) Tween 20. The ZPHL/HHL ratio for ACE in solution in the presence of the inhibitor (red bar in the right column) is shown for comparison. Data are expressed as a % of control (i.e. ACE without inhibitor) and presented as a mean of at least 3 independent experiments. The coloring here and in other figures is as follows: Values increased more than by 20% are highlighted in orange, more than 50% are highlighted in dark orange, more than by 100% are highlighted in red. Values decreased more than by 20% are highlighted in yellow.

A great advantage of this approach is a possibility to measure ACE activity in plasma taken with EDTA or in plasma containing ACE inhibitors, because EDTA or ACE inhibitors are washed out during washing step with distilled water with Tween-20 while ACE is still bound to mAbs. We determined the number of washings necessary to eliminate strong specific ACE inhibitor, enalaprilat, from the complex with ACE. It is worth noting that, while a compound can be a strong ACE inhibitor in physiological conditions, its binding constant to ACE significantly decreases in the absence of chloride [22], in this case, in distilled water. We measured ACE activity precipitated by 4 different mAbs using two substrates (ZPHL and HHL) and used the ratio of their rates of hydrolysis, ZPHL/HHL ratio, as a sensitive read-out of the presence of an inhibitor in complex with ACE [15, 23]–Fig 2. We found that the residual levels of ACE inhibitor in ACE precipitated by different mAbs are different, which is reflected in different ZPHL/HHL ratios at 1–3 washings, but five washings were enough for the complete dissociation of enalaprilat from the complex with ACE precipitated by any mAb (Fig 2).

**Fig 3:**
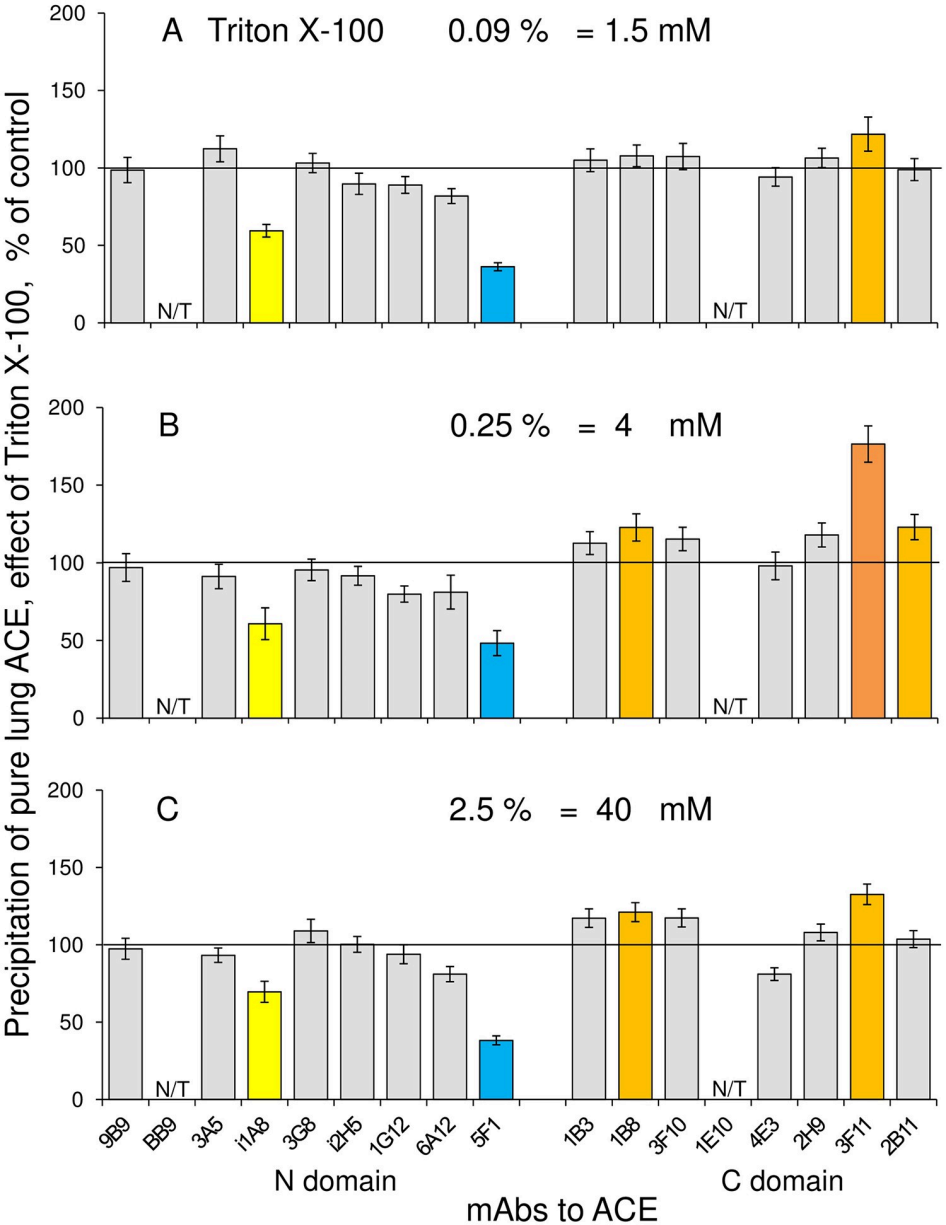
Effect of TritonX-100 on the mAbs binding to purified lung ACE. A-C. The effect of different concentrations of detergent Triton X-100 on the precipitation of ACE activity from ACE purified from human lung homogenate. Data are expressed as a % of control (i.e. without Triton X-100) and presented as a mean of at least 3 independent experiments. N/T–not tested mAbs. The coloring is as in the legend to Fig 2, and, in addition, values decreased more than by 50% are highlighted in deep blue.

**Fig 4:**
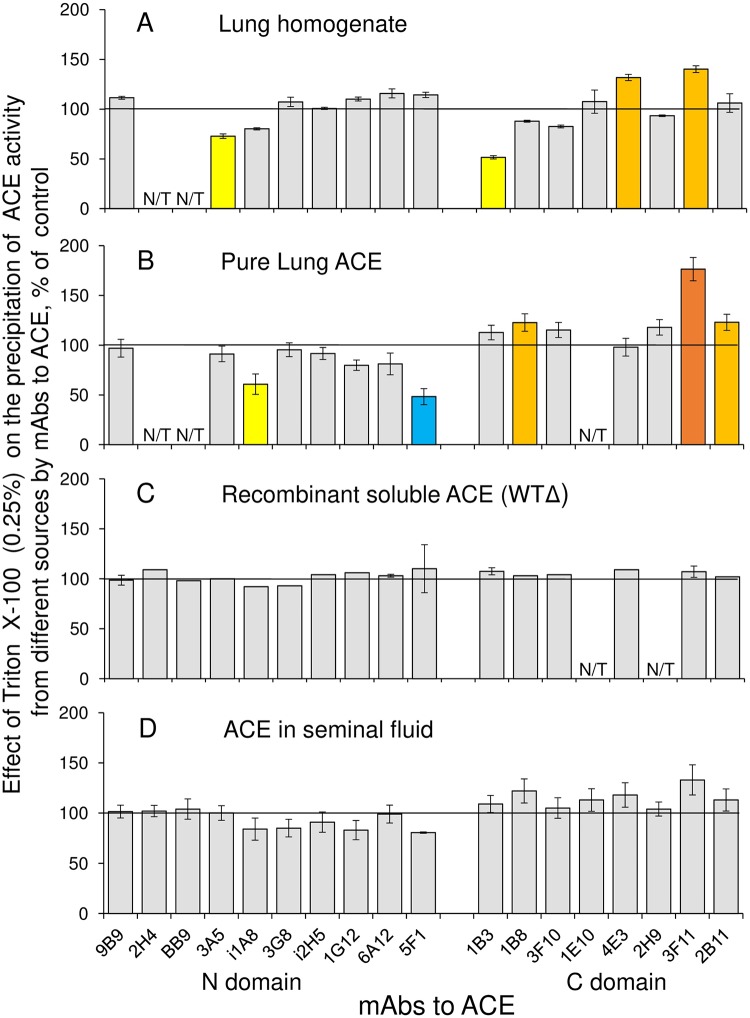
Effect of 0.25% Triton X-100 on mAbs binding to different ACEs. Data are expressed as a % of control (i.e., the source of ACE without Triton X-100) and presented as a mean of at least 3 independent experiments. N/T–not tested mAbs. The coloring is as in the legend to Fig 3.

Previously we found that the pattern of ACE activity precipitation by a set of mAbs—conformational fingerprint of ACE—could be sensitive to the presence of detergents [5]. Because we actively used this approach for the study of the fine conformation of ACE from different human tissues—tissue ACE specificity [9–10, 21]–we studied the effect of Triton X-100, used for ACE solubilization, on ACE conformational fingerprint. Fig 3 demonstrates the effect of different concentrations of Triton X-100 on ACE purified from human lung homogenate. It appeared that the binding of 4 mAbs to purified ACE was sensitive to the presence of the detergent—two mAbs, i1A8 and 5F1, to different epitopes on the N domain and two mAbs, 1B8 and 3F11, to the epitopes on the C domain (Fig 3). Intuitively, an effect of detergent on mAbs binding to ACE should be bigger with membrane form of ACE, i.e., with the enzyme containing transmembrane anchor, than with soluble ACE. We have shown previously [24] that even purified membrane form of ACE from bovine lung carried significant amount of Triton X-100. So, we tested an effect of Triton on mAbs binding with different ACEs and found that, indeed, while the effect of Triton was clearly seen with lung ACE, in crude homogenate or purified enzyme (Fig 4A and 4B), the presence of Triton did not influence on mAbs binding to soluble ACEs, recombinant ACE (Fig 4C) or ACE from seminal fluid (Fig 4D).

**Fig 5:**
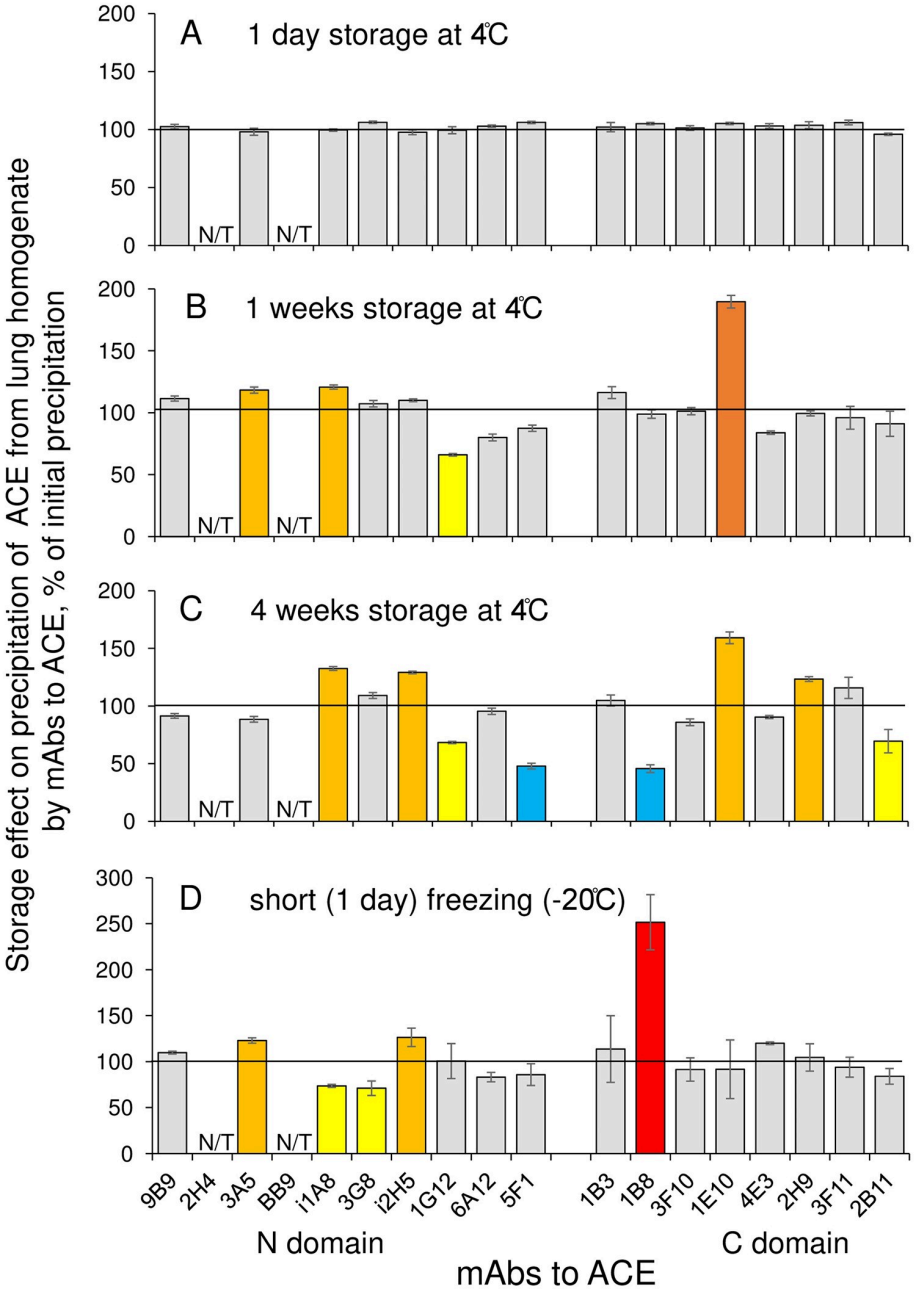
Effect of storage of lung homogenate on the recognition of ACE by mAbs. Freshly prepared human lung homogenate was prepared and then aliquoted into individual volumes for storage. Precipitation of ACE activity was compared for freshly prepared homogenate and the same homogenate but stored for different time and at different temperatures. A-C. Storage of homogenate at 4°C for different periods of time. **D**. Short freezing at -20°C. Data are expressed as a % of control (i.e., initial precipitation of ACE from freshly prepared lung homogenate) and presented as a mean of at least 3 independent experiments. N/T–not tested mAbs. The coloring is as in the legend to Fig 3.

Storage of human tissue homogenates can also alter ACE properties. We found that while ACE activity (with ZPHL as a substrate) in human lung homogenate was quite stable during up to 4 weeks of storage in a refrigerator, storage in a freezer at -20°C longer than one month resulted in a complete loss of ACE activity in human tissue homogenates or lysates of ACE expressing cells (not shown). The binding of some mAbs to ACE in human lung homogenate, however, noticeably changed already after one week of storage in a refrigerator and also changed even after short freezing (Fig 5). These data indicate on the changes of the topography of the surface of ACE protein globule during storage while active centers located deeply inside the protein globule [25] maintain their enzymatic functions. This observation is in accordance with our previous finding [26] by second derivative UV spectra of ACE solutions that γ-irradiation resulted in a decomposition of tyrosine and tryptophan residues on the surface of bovine ACE occurring even at low doses, while the enzyme retained its full enzymatic activity.

Thus, data presented in Figs 3–5 demonstrate that the fine changes in the surface topography of ACE from different sources should be measured in identical (or similar) conditions bearing in mind the presence of membrane anchor in some ACE molecules, as well as the conditions of ACE solubilization and storage.

### Detection of ACE inhibitors in patient’s blood

**Fig 6:**
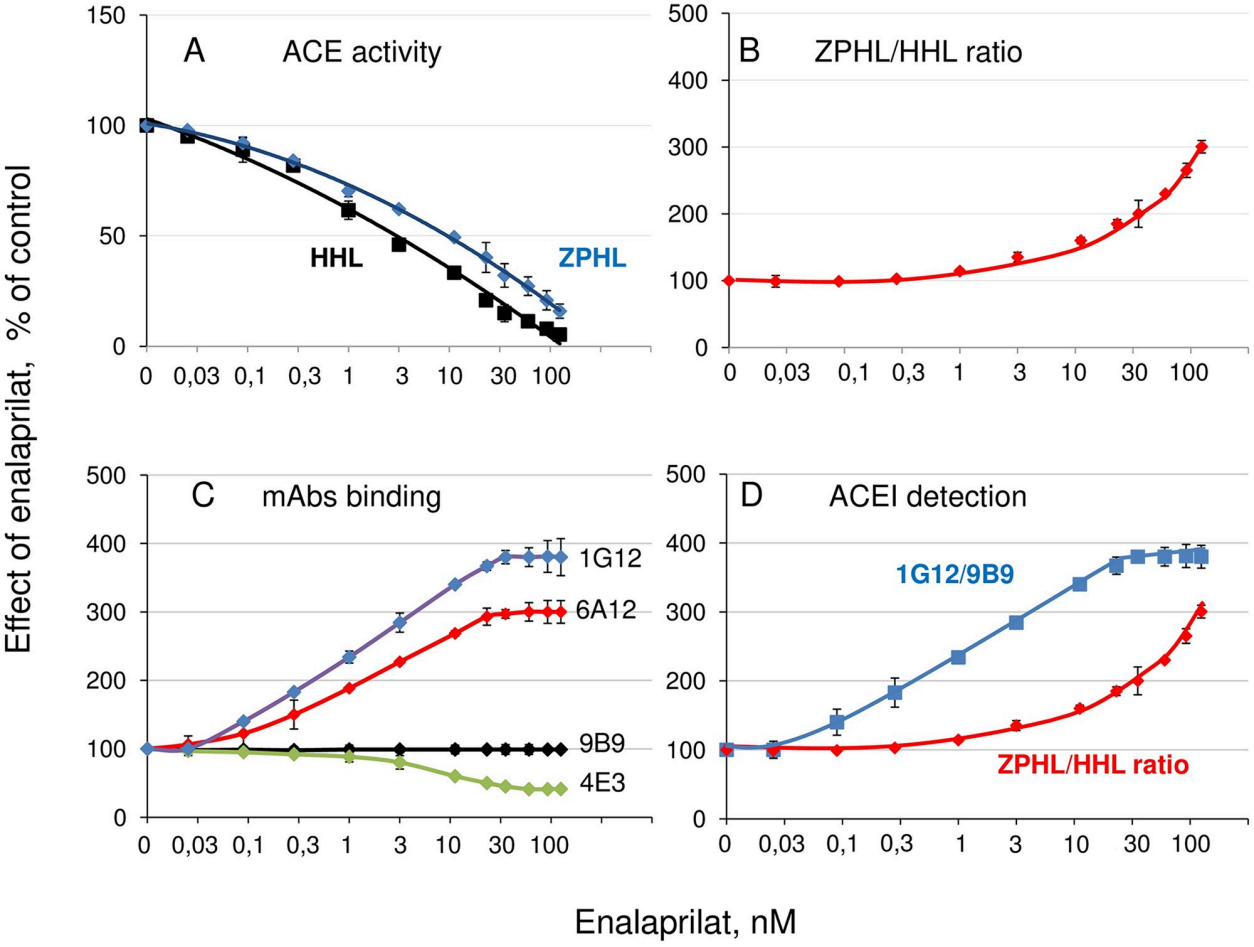
The detection of ACE inhibitors in the plasma of patients. Two different approaches for the detection of ACE inhibitors in the plasma of patients were compared using pooled citrated plasma from 80 patients not taking ACE inhibitors. The samples of plasma were spiked with different concentrations of enalaprilat. **A-B**. Enzymatic approach. **A**. ACE activity was determined by the rates of hydrolysis of two substrates, HHL and ZPHL, at different concentrations of ACE inhibitor enalaprilat, % from initial ACE activity. **B**. The ratio of the rates of the hydrolysis of substrates HHL and ZPHL, ZPHL/HHL ratio, determined in the presence of different enalaprilat concentrations, % from the value of the ratio in the absence of the inhibitor. **C**. Antibody-based assay: the efficacy of mAbs binding to ACE at different enalaprilat concentrations, % from the binding in the absence of the inhibitor. **D**. The comparison of enzymatic and mAbs-based methods. The effects of different concentrations of enalaprilat on two parameters, the ratio of the binding of mAbs 1G12 and 9B9 to ACE, 1G12/9B9, and ZPHL/HHL ratio. Data are expressed as a % of control (i.e., without ACE inhibitor enalaprilat) and presented as a mean of at least 3 independent experiments ± SD.

Previously we demonstrated the possibility of the detection of commercial ACE inhibitors in the patient’s blood using two different approaches—calculation of the ratio of the rates of the hydrolysis of two substrates, ZPHL/HHL ratio [15], and calculation of the ratio of the binding of two mAbs, 1G12 (or 6A12) and 9B9, to ACE [6], both ratios increasing in the presence of ACE inhibitors. The detection and quantification of ACE inhibitors in the patient’s blood (i.e. anti-hypertensive adherence) definitively has clinical significance, because patients with effective suppression of ACE has better blood pressure control than those with weak response to inhibitors [27]. Bearing this in mind, we accurately compared the sensitivity of both methods (Fig 6) and demonstrated that one of common ACE inhibitors, enalaprilat, could be detected in the patient’s blood at 1 nM using ZPHL/HHL ratio (Fig 6B and 6D) and even at less than 0.1 nM (Fig 6C and 6D) using mAb-based approach. For reference, the concentration of enalaprilat in the blood at its peak (4 hours after oral administration of 10 mg of enalapril) was reported to be about 50 nM [28]. Thus, even “restricted” conformational fingerprinting of ACE with only two mAbs allows obtaining valuable information.

### Conformational fingerprint of blood ACE from different donors

**Fig 7:**
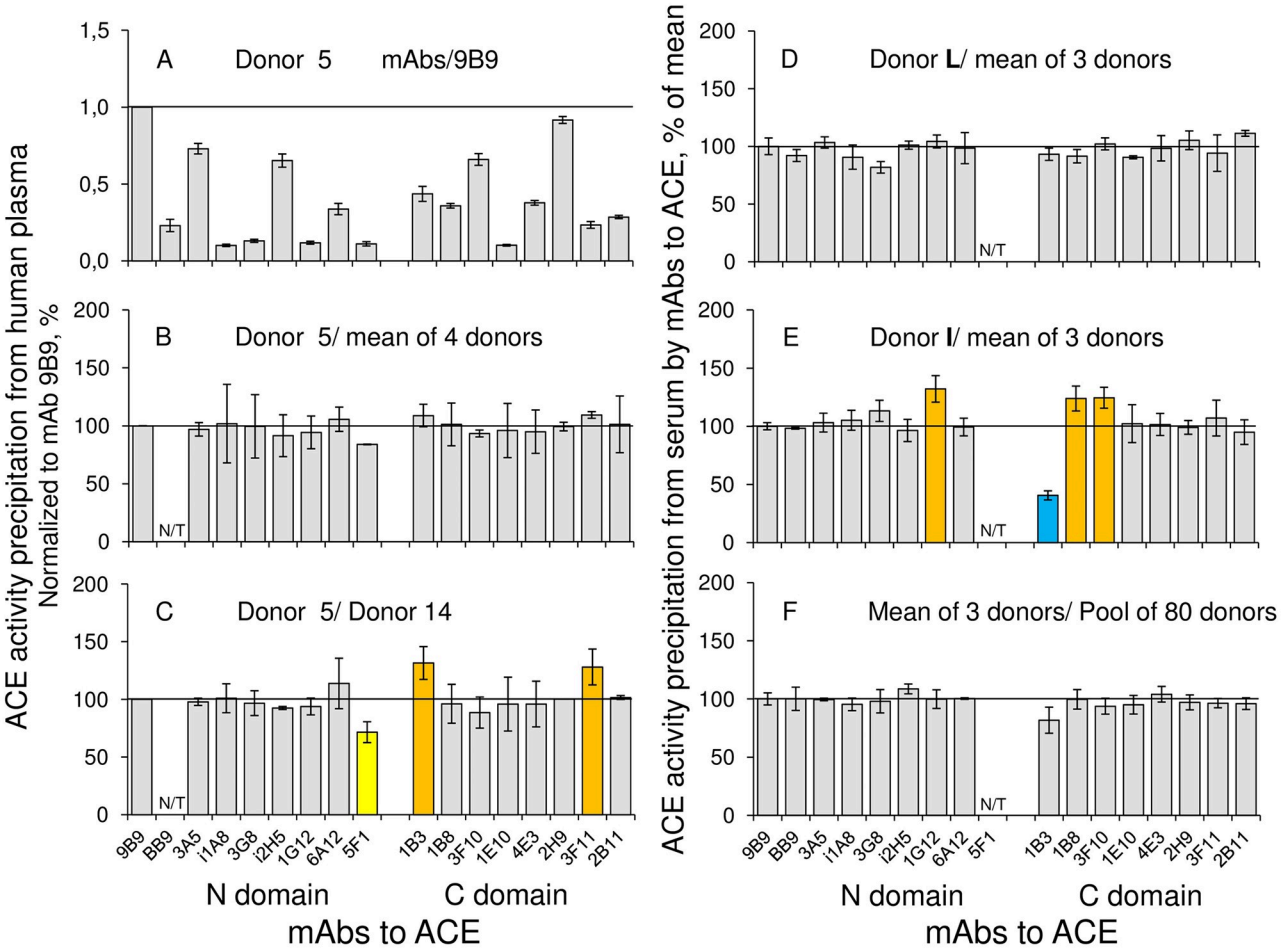
Comparative binding of mAbs to ACE from different donors. Conformational fingerprinting of ACE in plasma from donors with a set of mAbs to ACE. **A**. mAb/9B9 ratio for one of the donors, 5^th^ donor. **B**. The ratio of mAbs binding to ACE in plasma of 5^th^ donor to that for the mean for four donors. **C**. The ratio of precipitated activity of ACE in plasma of 5^th^ donor to that of 14^th^ donor is presented in order to highlight differences in immunoprecipitation pattern (“conformational fingerprint”) among ACE from blood of different donors. **D,E**. The ratio of mAbs binding to ACE in serum samples from two different donors to that for the mean for three donors. **F**. The ratio of mAbs binding to ACE in pooled serum samples from three donors to that for ACE pooled from 80 samples of normal plasma. Data for mAbs binding to ACE from all donors were preliminary normalized to the binding of the most strong mAb 9B9 to ACE from the same donor (as in A). The values obtained were used for the calculation of the ratios between different donors, expressed in % (B-F) and presented as a mean of at least 3 independent experiments. N/T–not tested mAbs. The coloring is as in the legend to Fig 3.

We quantified the precipitation of ACE activity from the plasma samples from different donors using wells coated (via goat-anti-mouse bridge) with 17 different mouse anti-ACE mAbs (Fig 7). Relative precipitation of ACE by strong mAbs (9B9, 2B11, 3A5, 2H9) dramatically differed compared to precipitation by weak mAbs (i1A8, 3G8, 1E10, and 3F11), likely reflecting difference in their affinity to ACE (Fig 7A). We should mention that some mAbs demonstrated anti-catalytic effect on ACE in solution at μg/ml concentrations [6, 15], whereas in the enzyme immunocapture assay (at an order lower concentrations the anti-catalytic effect did not exceed 20% (S1 Fig in [7]) in comparison with 10-fold differences in fluorescence signals demonstrated by ACE precipitated by different mAbs, weak and strong (Fig 7A). Therefore, we can consider anti-catalytic effect of some mAbs in this format as negligible.

We performed such immuno-capture assay with 8 randomly chosen plasma samples from healthy donors in two separate experiments with 5 and 3 donors. In order to see the putative inter-individual differences in the pattern of anti-ACE mAbs binding we presented the results as a ratio of ACE precipitation by any mAb (normalized for binding of mAb 9B9 to ACE corresponding to the level of the enzyme in plasma) for a certain donor to the mean value of ACE precipitation by this mAb for a number of donors (Fig 7B, 7D and 7E). The conformational fingerprint of blood ACE in general is very stable characteristics of the enzyme, as the pattern of mAbs binding to ACE from the pool from 3 plasma samples fairly well coincided with the pattern obtained for pool from 80 other plasma samples (Fig 7F). This analysis did not also reveal any peculiar ACE from blood of almost all patients, e.g., patients #5 and #L (Fig 7B and 7D), but showed that conformational fingerprint of ACE from the blood of patient #I differed from that for other donors (Fig 7E). Moreover, if to compare conformational fingerprint in pairs with more samples, it is possible to catch some differences in ACE conformation between individuals as well. Thus, we revealed such difference for ACE from patient #5 and patient #14 (Fig 7C), likely reflecting small inter-individual differences in ACEs from the blood of different donors.

It should be emphasized, that the effect of ACE inhibitors on mAbs binding (i.e., conformational changes in ACE) was much more prominent with up to 4-fold increase of the binding of mAbs 1G12/6A12 (Fig 6C and 6D), than inter-individual differences in ACE conformation which, in our experiments, did not exceed 30–40%. Nevertheless, the inter-individual differences observed were statistically significant (p<0.05) and reliable. While the clinical relevance of such inter-individual differences awaits further investigation, however, it could lead to the different behavior of the enzyme in some physiological processes, e.g. those including ACE interplay with effectors [7, 8] in blood and tissues.

### Conformational fingerprint of tissue ACE from different donors

**Fig 8:**
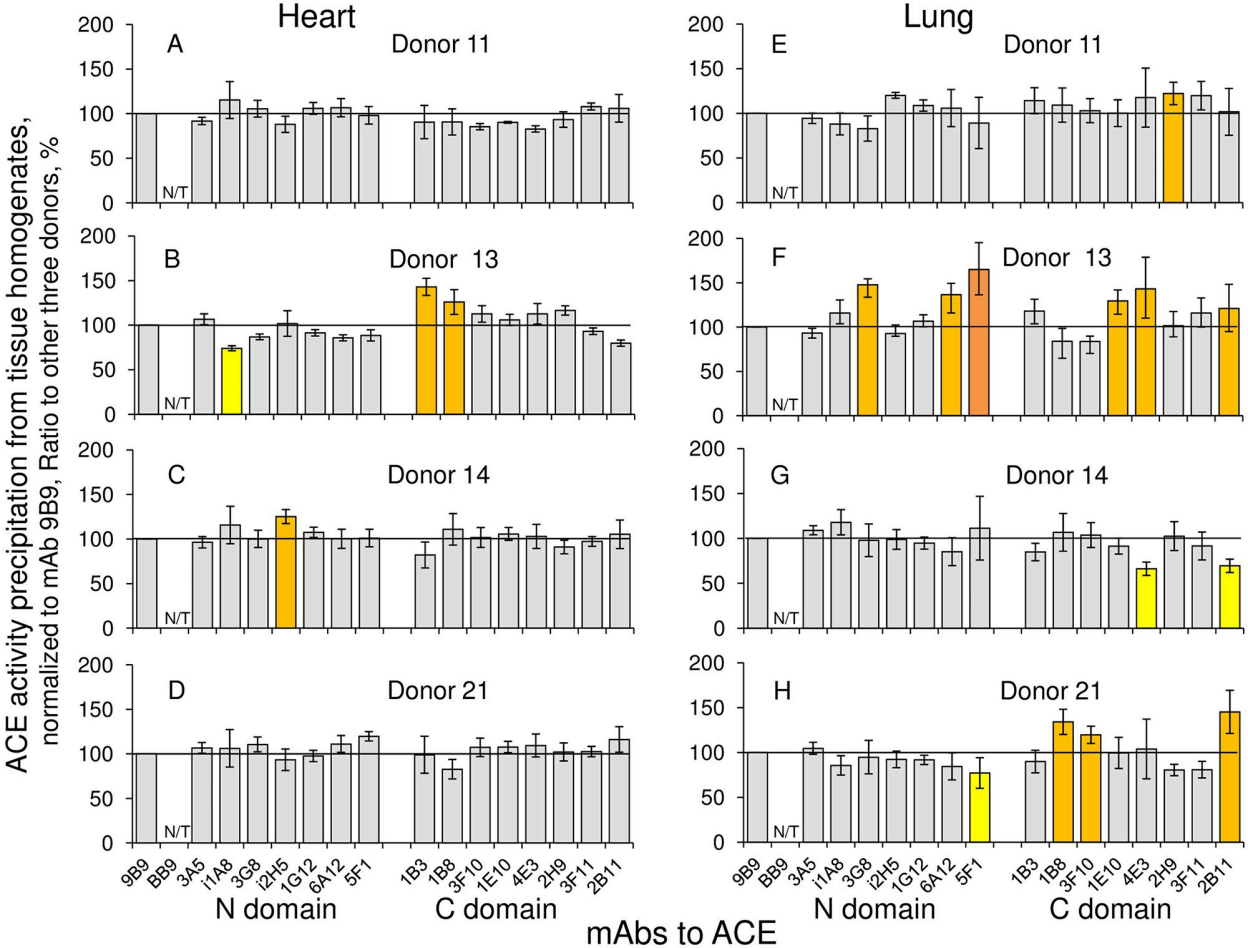
Comparative binding of mAbs to ACE from different tissues. Conformational fingerprinting of ACE in homogenates of heart and lung tissues from different donors with a set of mAbs to ACE. **A-D**. The ratio of immunoprecipitated ACE activity from heart homogenate of 11^th^, 13^th^, 14^th^, and 21^st^ donor to that for other three donors. **E-H**. The ratio of immunoprecipitated ACE activity from lung homogenate of 11^th^, 13^th^, 14^th^, and 21^st^ donor to that for other three donors. Data are normalized as in the legend to Fig 7 (to 9B9 precipitation), the ratios are expressed in % and presented as a mean of at least 3 independent experiments. N/T–not tested mAbs. The coloring is as in the legend to Fig 3.

Inter-individual differences in ACE conformation are more prominent for tissue ACEs. As an example, we show conformational fingerprints of heart and lung ACEs from different donors (Fig 8). Previously, we demonstrated that conformational fingerprint of ACE is very tissue-specific [9–10, 12] and the pattern of the binding of mAbs to ACE from heart (9 samples from different donors) remarkably differed from the pattern obtained for ACE from lung samples from the same donors. However, if to compare conformational fingerprint of ACE using more samples, it is possible to find some differences in ACE surface topography between individuals, as, for example, for donor #13 (Fig 8B and 8F) and other donors. Moreover; these differences could be tissue-specific, as seen for heart and lung ACEs (Fig 8).

**Fig 9:**
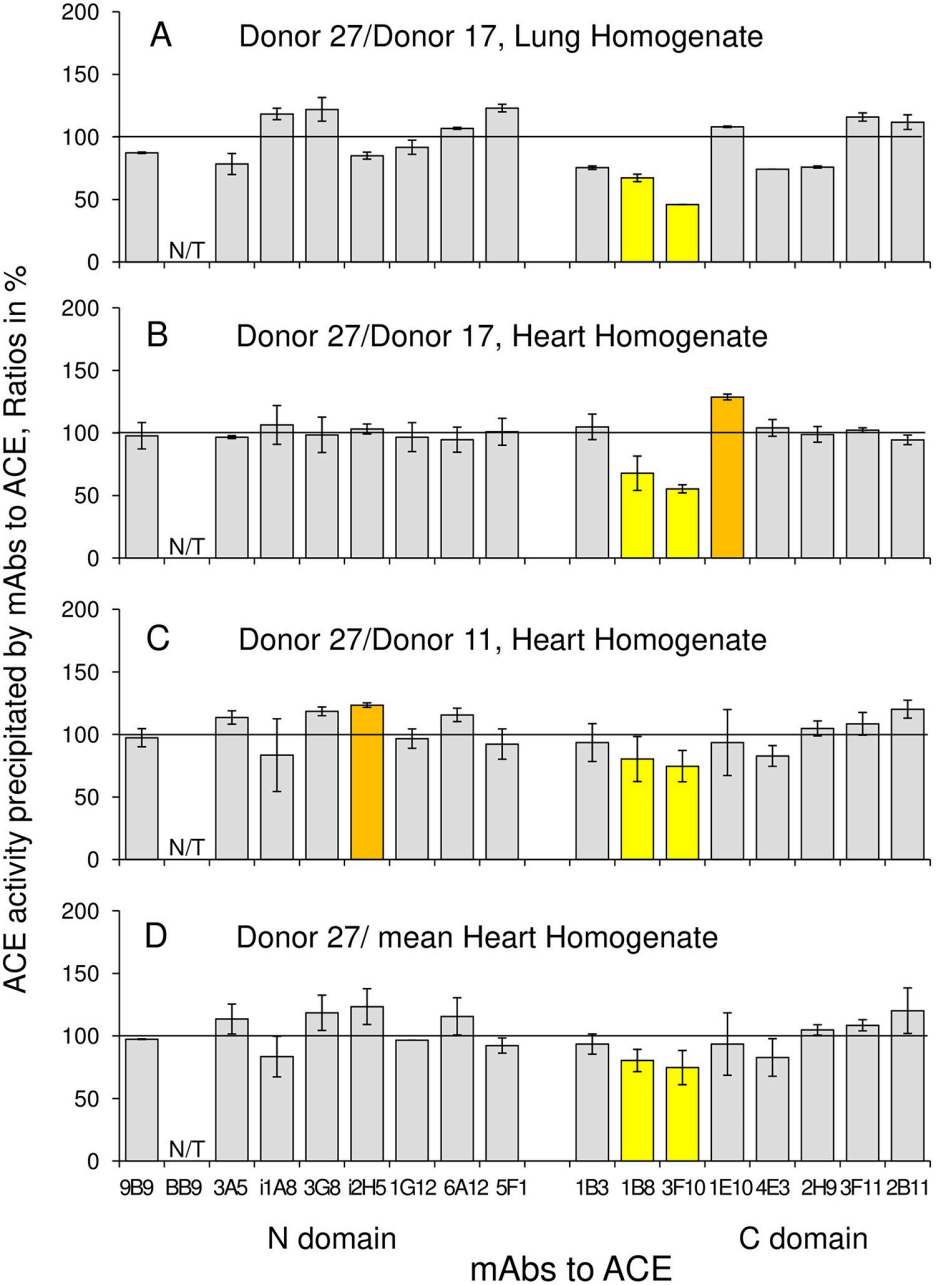
Inter-individual differences of the conformation of ACE from heart and lung. The ratios of precipitated ACE activity from any tissue homogenate to precipitated ACE activity from another homogenate is shown for clarity. **A**. The ratio of precipitated ACE activity from lung homogenate of 27^th^ donor to that of 17^th^ donor. **B**. The ratio of precipitated ACE activity from heart homogenate of 27^th^ donor to that of 17^th^ donor. **C**. The ratio of precipitated ACE activity from heart homogenate of 27^th^ donor to that of 11^th^ donor. **D**. The ratio of precipitated ACE activity from heart homogenate of 27^th^ donor to that for mean from 17^th^ and 11^th^ donor. Data are expressed in % and presented as a mean of at least 3 independent experiments. N/T–not tested mAbs. The coloring is as in the legend to Fig 3.

Conformational fingerprint of ACE from tissues of another donor, donor #27, revealed significant decrease in binding of two mAbs, 1B8 and 3F10 (Fig 9), which was observed when conformational fingerprints of ACE were compared in pairs (Fig 9A–9C), as well when conformational fingerprint of donor #27 was compared with that for mean from two other donors (Fig 9D). As the differences in 1B8/3F10 binding were found for ACEs from different tissues, heart and lung, we suspected a mutation in ACE in the overlapping region of the epitopes for mAbs 1B8/3F10. However, when we sequenced 9 exons of the C domain which coded the overlapping region of the epitopes for mAbs 1B8 and 3F10 [29], we did not find any ACE mutation that could be responsible for this phenotype. Moreover, we did not identify any known polymorphic variants of ACE gene in patient #27, but, at the same time, we identified silent single nucleotide polymorphism rs4331 in heterozygous state (AG) in patient #11, which does not lead to amino-acid substitution but may be associated with higher activity of ACE [30].

It is worth noting that tissue- and cell-specific post-translational modifications (PTM) are common for various proteins [31–32], which can subtly or dramatically alter the protein surface topography. The most common PTM is glycosylation. The inter-individual heterogeneity of the N-glycome is already reported, at least for plasma [33–34]. Therefore, it is possible that the mutation in one of the genes (out of more than 100 genes) responsible for glycosylation of the proteins in human [35] is a reason for the increase in 1B8/3F10 to ACE from patient #27.

Somatic ACE is a N-type glycoprotein characterized by a considerable content of its sugar moiety, e.g., human kidney ACE was reported to have about 18% of sugars [36]. The sequence of human somatic ACE contains 17 potential sites for N-glycosylation [3], however, the information about the structure and exact positions of glycan chains in ACE from different tissues is very limited [10, 12, 37–38]. The main part of glycans in somatic ACE belongs to the biantennary complex type oligosaccharides as well as high-mannose and hybrid type oligosaccharides [39]. These types of oligosaccharides possess the largest structural variation due to different possible amounts of the outer chains linked to the trimannosyl core and different structures of these chains. Besides, the outer chains can bear different amounts of neuraminic acid residues on their ends.

Almost all conformational epitopes for mAbs that we generated to the catalytically active human lung ACE contain potential glycosylation sites [5]. In particular, epitopes for mAbs 1B8 and 3F10 contain glycosylation site Asn731 [29]. Therefore, we can expect that it is glycan in glycosylation site Asn731 which is different in ACE from donor #27, whereas we could not also exclude another PTM for this donor at the moment.

**Fig 10:**
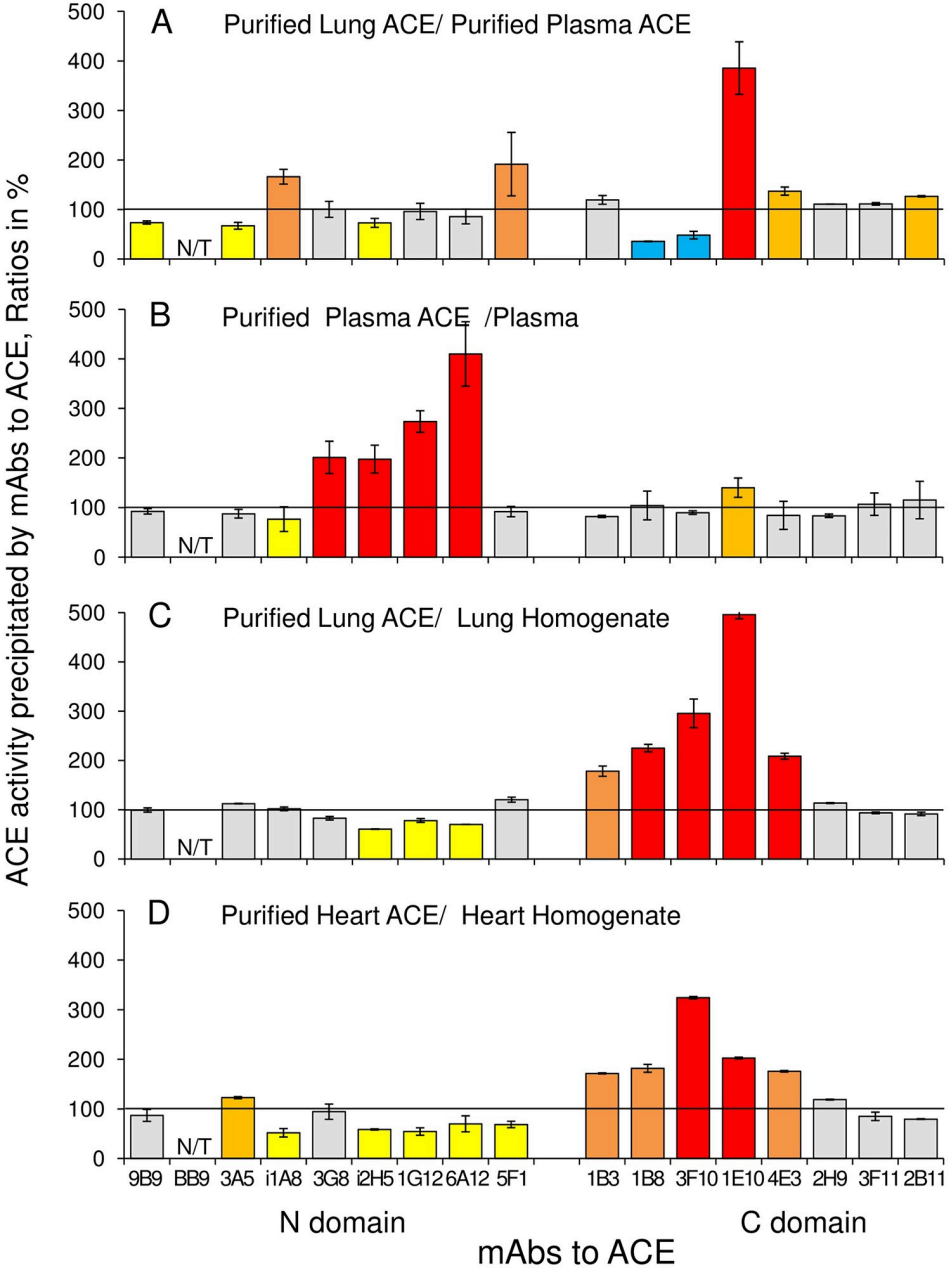
Effect of purification on the binding of mAbs to different ACEs. ACE activity in any pair of tested sources of ACEs was equilibrated with ZPHL as a substrate and precipitation of ACE activity was performed as in Fig 3. **A**. The ratio of precipitated activity of pure lung ACE to that of pure plasma ACE. **B**. Effect of ACE purification from human plasma on mAbs binding. **C** Effect of ACE purification from human heart homogenate (1:9, tissue:buffer) on mAbs binding. **D**. Effect of ACE purification o from human lung homogenate (1:9, tissue:buffer) on mAbs binding. Ratios are expressed in % and presented as a mean of at least 3 independent experiments. N/T–not tested mAbs. The coloring is as in the legend to Fig 3.

Further, an analysis of the pattern of a set of mAbs binding to ACE from different tissues and cells can provide some information about differences in glycosylation of ACE in these tissues [5, 9–10, 12], in particular, in sialylation of definite glycans. Fig 10A demonstrates the differences in the patterns of binding of mAbs to ACEs purified from the lung homogenate and from the blood plasma, i.e., the differences in the local surface topography of ACEs from different sources. Plasma ACE originates mainly from the lung capillaries (by 75%, according to our estimation [unpublished]) based on heterogeneous ACE expression in the capillaries from different organs [40]. It is known that sialic acid-deficient glycoproteins can be selectively removed from serum by macrophages and hepatocytes in the liver via lectins specifically recognizing penultimate galactose residues in oligosaccharide chains of a glycoprotein [41]. As an example, ACE from rabbit serum contains 3-fold higher molar ratio of sialyl- to galactosyl- residues than lung ACE [42]. Therefore, higher binding of mAbs i1A8 and 5F1 and much lower binding of mAbs 1B8 and 3F10 to lung ACE compared to plasma ACE (Fig 10A) could be attributed to the different sialylation of the oligosaccharide chains present in the epitopes of the corresponding mAbs. In particular, we can conclude that mAbs 1B8 and 3F10 to the C domain of ACE bind much better to the enzyme when glycan in the potential glycosylation site Asn731 is sialylated. The less effective binding of mAbs 1B8 and 3F10 to ACE from donor #27, therefore, indicates on less extent of sialylation (or even the absence of sialic acid residues) of this particular glycan in glycosylation site Asn731 in ACE from this donor compared to ACEs from other donors. Thus, conformational fingerprint can help to reveal some fine characteristics of the enzyme from a definite individual.

Besides, from the same data (Fig 10A) it is seen that mAb 1E10 to the C domain of ACE and mAbs 5F1 and i1A8 to the N domain bind better to the enzyme when oligosaccharide chains in the corresponding glycosylation sites, Asn666, Asn117 and Asn82, respectively, are not or, at least, less sialylated. This finding could be further extrapolated to the analysis of conformational fingerprint of ACE from other donors.

### Detection of ACE effectors in blood and tissue homogenates

Conformational fingerprinting of ACE can also provide valuable information on putative ACE effectors and ACE-binding proteins in the blood or different tissues. The influence of these effectors on the access of epitopes on the surface of ACE for mAbs is seen from the comparison of the conformational fingerprints of ACEs purified from lung and heart homogenate and from human plasma (Fig 10). Purification of ACE from human plasma resulted in an increase of binding of four mAbs (and decrease of one mAb) the N domain and one mAb to the C domain (Fig 10A). Purification of ACE from human heart decrease binding of five mAbs to the N domain, while one mAb to the N domain and five mAbs to the C domain increased their binding (Fig 10B). Purification of ACE from lung decreased binding of 3 mAbs to the N domain and increased binding of 5 mAbs to the C domain (Fig 10C).

From previous studies, we know that common ACE inhibitors, when bind to ACE, significantly increase the binding of two mAbs, 1G12 and 6A12, to the N-domain and decrease the binding of two anticatalytic mAbs, 1E10 and 4E3, to the C domain of ACE [6–8, 29]. Thus, the decrease in the binding of 1G12/6A12 mAbs and the increase in the binding of 1E10/4E3 mAbs with heart and lung ACEs could be attributed, at least partly, to the removal of some endogenous ACE inhibitors, accompanying ACE in the heart and lung, as a result of ACE purification. However, the significant increase in 1B3, 1B8 and 3F10 mAbs binding cannot be explained just by the removal of ACE inhibitors but rather by the removal of some other ACE effectors/ACE binding proteins accompanying ACE in the heart and lung and shielding (masking) epitopes for mAbs 1B3, 1B8/3F10, 1E10/4E3.

The strikingly different effect of the enzyme purification on its conformational fingerprint was obtained for plasma ACE. Namely, purification of ACE from human plasma resulted in the dramatic increase in binding of 4 mAbs to the overlapping region on the N domain, 1G12/6A12/i2H5 [6], and 3G8 [20], which we should explain mainly by the dissociation of bilirubin from its complex with plasma ACE as a result of the enzyme purification, as we have shown that bilirubin dramatically decreases the binding of these very mAbs [8]. The increase of mAb 1E10 binding to the C domain of plasma ACE after purification can be explained either by dissociation of endogenous inhibitors or/and by dissociation of lysozyme which forms complexes with ACE via binding to the cleft between ACE domains and to the region of 1E10 epitope [8].

Therefore, the conformational fingerprinting of ACE in different human tissues (using a set of mAbs to different epitopes of human ACE) provides a novel structural information about characteristics of ACE in different individuals (and in different tissues), as well as its effectors, that could be clinically important.

## Abbreviations

ACE: angiotensin-converting enzyme
Gal: galactose
HHL: hippuryl-L-histidyl-L-leucine
LMW: low molecular weight
PCR: polymerase chain reaction
PTM: post-translational modifications
SNP: single nucleotide polymorphism
ZPHL: carbobenzoxy-L-phenylalanyl-L-histidyl-L-leucine

## References

[1] Sturrock ED, Anthony CS, Danilov SM (2012) Peptidyl-dipeptidase A/Angiotensin I-converting enzyme In: Rawlings ND; Salvesen G, ed. Handbook of Proteolytic Enzymes, 3rd edn Oxford, Academic Press: 480–494.

[2] Bernstein KE, Ong FS, Blackwell WL, Shah KH, Giani JF, Gonzalez-Villalobos RA, et al (2013) A modern understanding of the traditional and nontraditional biological functions of Angiotensin-converting enzyme. Pharmacol Rev 65: 1–46. 10.1124/pr.112.006809 23257181 PMC3565918

[3] Soubrier F, Alhenc-Gelas F, Hubert C, Allegrini J, John M, Tregear G, et al (1988) Two putative active centers in human angiotensin I-converting enzyme revealed by molecular cloning. Proc Natl Acad Sci USA 85: 9386–9390. 2849100 10.1073/pnas.85.24.9386 PMC282757

[4] Danilov SM, Gordon K, Nesterovitch AB, Chen Z, Castellon M, Popova IA, et al (2011) An angiotensin I-converting enzyme mutation (Y465D) causes a dramatic increase in blood ACE via accelerated ACE shedding. PLoS One 6: e25952 10.1371/journal.pone.0025952 21998728 PMC3187827

[5] Danilov SM, Balyasnikova IB, Danilova AS, Naperova IA, Arablinskaya E, Borisov SE, et al (2010) Conformational fingerprinting of the angiotensin-converting enzyme (ACE): Application in sarcoidosis. J Proteome Res 9: 5782–5793. 10.1021/pr100564r 20873814

[6] Balyasnikova IV, Skirgello OE, Binevski PV, Nesterovich AB, Albrecht RFI, Kost OA, et al (2007) Monoclonal antibodies 1G12 and 6A12 to the N-domain of human angiotensin-converting enzyme: fine epitope mapping and antibody-based detection of ACE inhibitors in human blood. J Proteome Res 6: 1580–1594. 17326675 10.1021/pr060658x

[7] Petrov MN, Shilo VY, Tarasov AV, Schwartz DE, Garcia JGN, Kost OA, et al (2012) Conformational changes of blood ACE in chronic uremia. PLoS ONE 7: e49290 10.1371/journal.pone.0049290 23166630 PMC3500299

[8] Danilov SM, Lünsdorf H, Akinbi HT, Nesterovitch AB, Epshtein Y, Letsiou E, et al (2016) Lysozyme and bilirubin bind to ACE and regulate its conformation and shedding. Sci Rep 6: 34913–34913. 10.1038/srep34913 27734897 PMC5062130

[9] Tikhomirova VE, Kost OA, Kryukova OV, Golukhova EZ, Bulaeva NI, Zholbaeva AZ, et al (2017) ACE phenotyping in human heart. PloS ONE, 12: e0181976 10.1371/journal.pone.0181976 28771512 PMC5542439

[10] Kryukova OV, Tikhomirova VE, Golukhova EZ, Evdokimov VV, Kalantarov GF, Trakht IN, et al (2015) Tissue specificity of human angiotensin I-converting enzyme. PLoS One 10: e0143455 10.1371/journal.pone.0143455 26600189 PMC4658169

[11] Danilov SM (2017) Conformational fingerprinting using monoclonal antibodies (on the example of angiotensin-converting enzyme. Mol Biol (Mosk) 51:1046–1061. 29271967 10.7868/S0026898417060155

[12] Kost OA, Tikhomirova VE, Kryukova OV, Gusakov AV, Bulaeva NI, Evdokimov VV, et al (2018) A conformational “fingerprint” of the angiotensin-converting enzyme. Russian J Bioorg Chem 44: 52–63.

[13] Kost OA, Grinshtein SV, Nikolskaya II, Shevchenko AA, Binevski PV (1997). Purification of soluble and membrane forms of somatic angiotensin-converting enzyme by cascade affinity chromatography. Biochemistry (Mosc) 62: 321–328. 9275304

[14] Wei L, Alhenc-Gelas F, Soubrier F, Michaud A, Corvol P, Clauser E. (1991) Expression and characterization of recombinant human angiotensin I-converting enzyme. Evidence for a C-terminal transmembrane anchor and for a proteolytic processing of the secreted recombinant and plasma enzymes. J Biol Chem 266: 5540–5546. 1848554

[15] Danilov SM, Balyasnikova IV, Albrecht RF II, Kost OA (2008) Simultaneous determination of ACE activity with two substrates provides information on the status of somatic ACE and allows detection of inhibitors in human blood. J Cardiovasc Pharmacol 52: 90–103. 10.1097/FJC.0b013e31817fd3bc 18645413

[16] Danilov SM, Jaspard E, Churakova T, Towbin H, Savoie F, Wei L, et al (1994) Structure-function analysis of angiotensin I-converting enzyme using monoclonal antibodies. J Biol Chem 269: 26806–26814. 7523412

[17] Jokubaitis VJ, Sinka L, Driessen R, Whitty G, Haylock DN, Bentoncello I, et al (2008) Angiotensin-converting enzyme (CD143) marks hematopoietic stem cells in human embryonic, fetal and adult hematopoietic tissues. Blood 111: 4055–4063. 10.1182/blood-2007-05-091710 17993616

[18] Dufour C, Casane D, Denton D, Wickings J, Corvol P, Jeunemaitre X (2000) Human-chimpanzee DNA sequence variation in the four major genes of the renin angiotensin system. Genomics 69: 14–26. 10.1006/geno.2000.6313 11013071

[19] Danilov SM, Allikmets EY, Sakharov IY, Dukhanina EA, Trakht IN (1987) Monoclonal antibodies to human angiotensin-converting enzyme. Biotech Appl Biochem. 9: 319–312. 2822061

[20] Gordon K, Balyasnikova IV, Nesterovitch AB, Schwartz DE, Sturrock ED, Danilov SM (2010) Fine epitope mapping of monoclonal antibodies 9B9 and 3G8, to the N domain of human angiotensin I-converting enzyme (ACE) defines a region involved in regulating ACE dimerization and shedding. Tissue Antigens 75: 136–150. 10.1111/j.1399-0039.2009.01416.x 20003136

[21] Danilov SM, Tikhomirova VE, Metzger R, Naperova IA, Bukina TM, Goker-Alpan O, et al (2018) ACE phenotyping in Gaucher disease. Mol Genet Metab 123: 501–510. 10.1016/j.ymgme.2018.02.007 29478818 PMC5891352

[22] Bull HG, Thornberry NA, Cordes MH, Patchett AA, Cordes EH (1985) Inhibition of rabbit lung angiotensin-converting enzyme by N alpha-[(S)-1-carboxy-3-phenylpropyl]L-alanyl-L-proline and N alpha-[(S)-1-carboxy-3-phenylpropyl]L-lysyl-L-proline. J Biol Chem 260: 2952–2962. 2982845

[23] Danilov SM, Tovsky SI, Schwartz DE, Dull RO (2017) ACE Phenotyping as a guide toward personalized therapy with ACE inhibitors. J Cardiovasc Pharmacol Ther 22: 374–386. 10.1177/1074248416686188 28587581

[24] Grinshtein SV, Nikolskaya II, Klyachko NL, Levashov AV, Kost OA (1999) Structural organization of membrane and soluble forms of somatic angiotensin-converting enzyme. Biochemistry (Mosc) 64: 571–589. 10381620

[25] Bernstein KE, Welsh SL, Inman JK (1990) A deeply recessed active site in angiotensin-converting enzyme is indicated from the binding characteristics of biotin-spacer-inhibitor reagents. Biochem Biophys Res Commun 167: 310–316. 2155615 10.1016/0006-291x(90)91766-l

[26] Orlova MA, Kost OA, Nikolskaya II, Troshina NN, Shevchenko AA (1994) Radiation-induced inactivation of angiotensin-converting enzyme in aqueous solutions. I. The effect of irradiation conditions. Russian Chemical Bulletin 43: 1260–1264.

[27] Jones ESW, Lesosky M, Blockman M, Castel S, Decloedt EH, Schwager SLU, et al (2017) Therapeutic drug monitoring of amlodipine and the Z-FHL/HHL ratio: Adherence tools in patients referred for apparent treatment-resistant hypertension. S Afr Med J 107: 887–891. 10.7196/SAMJ.2017.v107i10.12268 29022534

[28] Gu Q, Chen X, Zhong D, Wang Y (2004) Simultaneous determination of enalapril and enalaprilat in human plasma by liquid chromatography—tandem mass spectrometry. J Chromatography B 813: 337–342. 10.1016/j.jchromb.2004.09.031 15556550

[29] Naperova IA, Balyasnikova IV, Schwartz DE, Watermeyer J, Sturrock ED, Kost OA, et al (2008) Mapping of conformational mAb epitopes to the C domain of human angiotensin I-converting enzyme (ACE). J Proteome Res 7: 3396–3411. 10.1021/pr800142w 18576678

[30] Chung CM, Wang RY, Fann CS, Chen JW, Jong YS, Jou YS, et al (2013) Fine-mapping angiotensin-converting enzyme gene: separate QTLs identified for hypertension and for ACE activity. PLoS One. 8(3):e56119 10.1371/journal.pone.0056119 23469169 PMC3587614

[31] Brogren H, Sihlbom C, Wallmark K, Lönn M, Deinum J, Karlsson L, et al (2008) Heterogeneous glycosylation patterns of human PAI-1 may reveal its cellular origin. Thromb Res 122: 271–281. 10.1016/j.thromres.2008.04.008 18508114

[32] Liddy KA, White MY, Cordwell SJ (2013) Functional decorations: post-translational modifications and heart disease delineated by targeted proteomics. Genome Med 5: 20 10.1186/gm424 23445784 PMC3706772

[33] Hennig R, Cajic S, Borowiak M, Hoffmann M, Kottler R, Reichl U, et al (2016) Towards personalized diagnostics via longitudinal study of the human plasma N-glycome. Biochim Biophys Acta 1860: 1728–1738. 10.1016/j.bbagen.2016.03.035 27038647

[34] Lauc G, Pezer M, Rudan I, Campbell H (2016) Mechanisms of disease: The human N-glycome. Biochim Biophys Acta 1860: 1574–1582. 10.1016/j.bbagen.2015.10.016 26500099

[35] Varki A, Cummings RD, Esko JD, Freeze HH, Stanley P Bertozzi CR, et al, editors) In: Essentials of Glycobiology. 2nd edition, Cold Spring Harbor (NY): Cold Spring Harbor Laboratory Press; 2009 20301239

[36] Ehlers MR, Chen YN, Riordan JF (1992) The unique N-terminal sequence of testis angiotensin-converting enzyme is heavily O-glycosylated and unessential for activity or stability. Biochim Biophys Res Commun, 183: 199–205. 10.1016/0006-291x(92)91628-4 1311921

[37] Yu XC, Sturrock ED, Wu Z, Biemann K, Ehlers MRW, Riordan JF (1997) Identification of N-linked glycosylation sites in human testis angiotensin-converting enzyme and expression of an active deglycosylated form. J Biol Chem 272: 3511–3519. 9013598 10.1074/jbc.272.6.3511

[38] Lui T, Qian W-J, Gritsenko MA, Camp DG 2nd, Monroe ME, Moore RJ, et al (2005) Human plasma N-glycoproteome analysis by immunoaffinity subtraction, hydrazide Chemistry, and mass spectroscopy. J Proteome Res 4: 2070–2080 10.1021/pr0502065 16335952 PMC1850943

[39] Ripka JE, Ryan JW, Valido FA Chung AY, Peterson CM, Urry RL (1993) N-glycosylation of forms of angiotensin converting enzyme from four mammalian species. Biochem Biophys Res Commun 196: 503–508. 10.1006/bbrc.1993.2278 8240320

[40] Metzger R, Franke FF, Bohle RM, Alhenc-Gelas F, Danilov SM (2011) Heterogeneous distribution of Angiotensin I-converting enzyme (CD143) in the human and rat vascular systems: vessels, organs and species specificity. Microvasc Res. 81: 206–215 10.1016/j.mvr.2010.12.003 21167844

[41] Varki A, Schauer R. Sialic Acids In: Essentials of Glycobiology. 2nd edition (Varki A, Cummings RD, Esko JD, Freeze HH, Stanley P, Bertozzi CR, Hart GW, Etzler ME, editors) Cold Spring Harbor (NY): Cold Spring Harbor Laboratory Press; 2009. Chapter 14. 20301239

[42] Das M, Hartley JL, Soffers RL (1997) Serum angiotensin-converting enzyme. Isolation and relationship to the pulmonary enzyme. J Biol Chem, 252: 1316–1319. 190228

